# ITGA5 and ITGB1 contribute to Sorafenib resistance by promoting vasculogenic mimicry formation in hepatocellular carcinoma

**DOI:** 10.1002/cam4.5110

**Published:** 2022-08-10

**Authors:** Ying Shi, Jin Shang, Yan Li, Deyuan Zhong, Zilong Zhang, Qinyan Yang, Chunyou Lai, Tianhang Feng, Yutong Yao, Xiaolun Huang

**Affiliations:** ^1^ Department of Hepatobiliary‐Pancreatic Surgery, Cell Transplantation Center, Sichuan Provincial People's Hospital University of Electronic Science and Technology of China Chengdu Sichuan China; ^2^ School of Medicine Chengdu Sichuan China; ^3^ Sichuan Translational Medicine Research Hospital, Chinese Academy of Sciences Chengdu Sichuan China

**Keywords:** drug resistance, hepatocellular carcinoma, hypoxia, integrin, sorafenib, vascular mimicry

## Abstract

**Background:**

Hepatocellular carcinoma (HCC) is labeled with high mortality and tolerance to chemotherapy. Sorafenib has been the first‐line treatment option in HCC patients for past decades, while the therapeutic effect was limited in almost HCC patients.

**Methods:**

In this study, we analyzed public omics data of HCC patients with different responses to Sorafenib treatment. To confirm the role of integrins A5 and B1 (ITGA5 and ITGB1) in Sorafenib resistance, we generated the Sorafenib‐resistant (Sor‐R) cell lines and cells overexpressing ITGA5 or ITGB1. Hypoxia level was measured using Hypoxy probe by flow cytometry, while vasculogenic mimicry was detected and quantified by CD31 and periodic acid schiff staining.

**Results:**

Hypoxia was upregulated in non‐responsive patients, accompanied with genes involved in encoding extracellular matrix components and angiogenesis such as ITGA5 and ITGB1. Sor‐R hepatoma cell lines were constructed to measure expression and role of candidate genes. ITGA5 and ITGB1 were augmented in Sor‐R cells. Upregulation of ITGA5 or ITGB1 reduced the sensitivity to Sorafenib in HepG2 and Huh7 cells, aggravated the hypoxic condition and resulted in formation of vascular mimicry.

**Conclusions:**

These findings suggested that hypoxia associated vascular mimicry account for non‐response to Sorafenib treatment in HCC patients. ITGA5 and ITGB1 may serve as effective predictors of HCC patients' outcome after Sorafenib treatment, which also provides a new target for HCC patients resistant to Sorafenib.

## Graphical abstract

Hypoxia associated vascular mimicry account for non‐response to Sorafenib treatment in hepatocellular carcinom (HCC) patients. integrins A5 and B1 may serve as effective predictors of HCC patients' outcome after Sorafenib treatment, which also provides a new target for HCC patients resistant to Sorafenib.

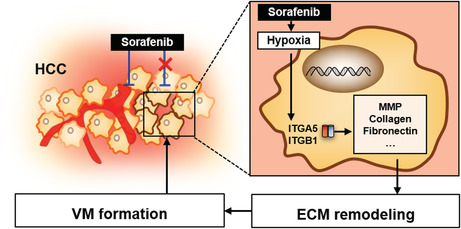

## 1 BACKGROUND

Primary liver cancer ranks the sixth of most frequently diagnosed cancer, becoming the third leading cause of cancer‐related death worldwide in 2020. The major types of primary liver cancer are hepatocellular carcinoma (HCC, 75%–85%) and intrahepatic cholangiocarcinoma (10%–15%).^1^ Standard therapy for HCC patients contains locoregional therapies (e.g., surgical resection, liver transplantation, ablation and transarterial chemoembolization) and systemic therapies (e.g., immunotherapy, chemotherapy, and some molecular‐targeted drugs).^2^ For the past decades, Sorafenib was the only first‐line drug approved by FDA toward advanced HCC patients.^3^ As an oral multi‐kinase tyrosine kinase inhibitor, Sorafenib elicits anti‐angiogenic and anti‐proliferation property by targeting on angiogenic or oncogenic factors, including v‐raf murine sarcoma viral oncogene homolog B1 (BRAF), platelet‐derived growth factor receptor beta, vascular endothelial growth factor receptors (VEGFR1, VEGFR2, VEGFR3). However, only 30% of HCC patients benefit from Sorafenib treatment, indicating preexisting innate or acquired resistance to Sorafenib in HCC patients based on accumulating results of clinical trials (e.g., NCT00105443 and NCT00492752).^4^

Mechanism of Sorafenib resistance has been intensively investigated by high‐throughput genome‐wide screening tools. Genes involved in hypoxia‐angiogenesis cascade were found to be crucial in attenuating efficacy of Sorafenib. HCC is characterized with high invasive ability and oxygen‐consuming, requiring abundant tumor vessels to provide oxygen and nutrients. Accordingly, fast‐growing tumor results in an intratumor hypoxic microenvironment, boosting a batch of angiogenic factors to promote neovascularization in an endothelial cells (ECs)‐dependent manner, such as vascular endothelial growth factor (VEGF) and angiopoietin as well as their receptors or ligands.^5^ Vessels originated from ECs carry endothelial markers, presenting with positive staining of CD31 platelet and endothelial cell adhesion molecule 1 (PECAM1) or CD34. Sorafenib is supposed to intercept key steps of tumor vessel formation, therefore inhibit tumor growth consequently. Notwithstanding, accumulating evidence unearthed that high heterogeneous intratumoral angiogenetic patterns greatly contributed to Sorafenib resistance, resulting in malignant phenotype and poor outcome in HCC patients.^6^

Compared to the classical ECs‐dependent vascular, those ECs‐independent or mosaic vessels account for reasonable quantity of tumor vessels.^7^ Vasculogenic mimicry (VM), an ECs‐independent pathway of tumor neovascularization, complementing oxygen and nutrients supply even when classical ECs‐original vessels are blocked.^8^ Under the hypoxia circumstances, sensors of hypoxia like hypoxia‐induced factors (HIF)‐1α enable tumor cells perceive reduced oxygen content, inducing extracellular matrix (ECM) re‐molding and epithelial‐mesenchymal transition (EMT) process subsequently.^9^ Besides with reflecting dynamic process of EMT, transition or change of major ECM components like collagens and integrins can affect cell polarity and greatly participated in VM formation. Different from ECs‐originated vessels, VM structure are positive in periodic acid schiff (PAS) staining but lack of CD31 or CD34. Integrins are heterodimeric transmembrane glycoproteins expressed on cell surface, acting essential role in mediating processes such as cell adhesion and migration. Once combining with their distinct ligands, an active conformational change can be triggered on integrins, endowing them with ability of bi‐directional intra‐ or intercellular signaling transduction.^10^ In this study, we found that HCC patients resistant to Sorafenib were featured with higher integrins A5 and B1 (ITGA5 and ITGB1) compared to non‐resistant patients. Further, we investigated role of ITGA5 and ITGB1 in Sorafenib‐resistance and integrin inhibitor (ATN‐161). This research is supposed to provide an explanation for Sorafenib resistance, contributing to further development of new target therapy in Sorafenib‐resistant (Sor‐R) HCC patients.

## 2 MATERIALS AND METHODS

### 2.1 Public data acquisition and analysis

The transcriptomic data and clinical information of HCC patients were downloaded from GEO database (https://www.ncbi.nlm.nih.gov/geo/; GSE109211) and the cancer genome atlas (TCGA database, https://portal.gdc.cancer.gov/).^11^ Differentially expressed genes (DEGs) were analyzed using data of GSE109211 in R platform by using “DESeq2” and “ggplot2” packages, with the definition of *p* < 0.05 and absolute log2FoldChange >1. Kyoto encyclopedia of genes and genomes (KEGG) analysis were performed using those DEGs, and differentially expressed pathways or symptoms were illustrated. For validation, the medication and clinical follow‐up information of HCC patients from TCGA database were integrated to perform Kaplan–Meier analysis by using the R package “survival”. For hypoxia evaluation and correlation analysis, a model based on the Buffa mRNA abundance signature were used to calculate the hypoxia score.^12^ The difference of hypoxia symptoms was illustrated using gene set enrichment analysis (GSEA) software. The Pearson correlation between hypoxia score and expression of genes were calculated using R package “ggplot2”. Multi‐Cox regression was selected to determine Hazard ratio of different genes by using the R package “survival”. Images of immunohistochemistry of HCC tissue were downloaded from the Human Protein Atlas (https://www.proteinatlas.org/).

### 2.2 Cell lines and cell culture

The HCC cell lines (HepG2 and Huh7) and lymphocyte (HEK‐293T) were purchased from American Type Culture Collection, certificated by short tandem repeat (STR). Cells were cultured as monolayers in DMEM medium with 10% fetal bovine serum and 1% antibiotics (penicillin and streptomycin) at 37°C in a humidified 5% CO_2_ atmosphere with the absence of Mycoplasma.

### 2.3 Reagents

Sorafenib (S7397, Selleck) and Ac‐PHSCN‐NH2 (ATN‐161) (S8454, Selleck) were solubilized in DMSO or sterile water with a concentration of 100 and 40 mM, respectively, stored at −20°C for further use. CoCl_2_ (232696, Sigma) was weighed and solubilized directly in culture media for use. RPMI‐1640 medium, antibiotics (penicillin and streptomycin) and fetal bovine serum utilized in this study were obtained from Gibco (Grand). Hypoxyprobe™ Plus Kit was acquired from (HP2‐100Kit; Hypoxyprobe Inc). Glycogen Periodic Acid Schiff Stain Kit was purchased from Solarbio (G1281). We obtained all other analytical grade reagents from Fisher Scientific and Sigma‐Aldrich and used them without further purification.

### 2.4 Western‐blot

Total proteins were extracted from whole cells and analyzed for expression of ITGA5, ITGB1, and GAPDH by Western blot assay. Total proteins were extracted and separated by sodium dodecyl sulfate‐polyacrylamide gel electrophoresis, and transferred onto PVDF membranes. Fat‐free milk of 5% was utilized in blocking for 1–3 h at room temperature and further incubated with ITGA5 (1:2500, 10569‐1‐AP, Proteintech), ITGB1 (1:2500, 12594‐1‐AP, Proteintech), HIF1A (1:1500, 66730‐1‐Ig, Proteintech) and GAPDH (1:4000, 60004‐1‐Ig, Proteintech) antibodies overnight at 4°C. Blots were then incubated with goat anti‐rabbit secondary antibody (1:4000) conjugated with horseradish peroxidase and finally imaged by chemiluminescence in working solution.

### 2.5 Lentiviral particles construction and cell transfection

ITGA5 and ITGB1 over‐expression plasmids were generated by inserting the human full length ITGA5 cDNA (NM_002205.5) and ITGB1 cDNA (NM_002211.4) into pLVX‐puro vector, respectively. Lentiviral viral particles suspended in culture media of HEK‐293 T cells infected with plasmids described above were collected. Viral particles were transfected in HepG2 and Huh7 cells; puromycin was used to select cells with stable lentiviral integrated.

### 2.6 Assessment of cell viability

The wild‐type of HepG2 and Hun7 were exposed to 1, 5, and 10 μM Sorafenib or Sorafenib combined with 100 μM ATN‐161 to investigate the 50% inhabitation concentration (IC_50_) after 48‐, 72‐h exposure through cell counting kit (CCK)‐8 assay. Cells were plated in 96‐well plates with a density of 2 × 10^4^ HepG2 cells/well and 1.5 × 10^4^ Huh7 cells/well overnight. After 48‐, 72‐h of incubation with Sorafenib and ATN‐161, cells were incubated with 10 μl CCK‐8 working solution for 4 h at 37°C in a humidified 5% CO_2_. After that, absorbance was measured at 450 nm wavelength. Growth inhibition curves were plotted as the percentage of untreated control cells.

### 2.7 Glycogen PAS staining

Cells seeded on slides underwent fixation with 10% formalin for 15 min were prepared for PAS staining following the procedure according to its instruction (Solarbio, G1280). Slides were treated with periodic acid solution and Schiff Reagent orderly. Next, slides were stained with staining hematoxylin solution, acidic differentiation solutions were used to remove excess background staining. The slides were then dehydrated, cleared for image collection on microscope (Olympus).

PAS staining was also performed in the 3D scaffolds culture to evaluate VM.^13^ The 3D scaffolds were soaked in DMEM for 8 h then sterilized filter papers were used to absorb DMEM. Cells were seeded on 3D scaffolds and cultured for 4 h before medium was supplemented. After 3‐day culture. Scaffolds with cells were cut into frozen sections, which were fixed to undergo PAS staining and observed as described above.

### 2.8 Cell hypoxic condition detection assay

An algorithmic model was used to access the hypoxia score in Sor‐R and Sorafenib‐non‐resistant (Sor‐NR) groups.^12^ Hypoxic level was measured through Hypoxyprobe™ Plus Kit according to instructions. Cells were plated into 6‐well plates with a density of 2 × 10^4^ HepG2 cells/well and 1.5 × 10^4^ Huh7 cells/well overnight. After 24 h, cells were incubated with 200 μM Pimonidazol HCl and dissociated to collection. Cells underwent centrifuge were then resuspended and incubation with Fixation Buffer and Permeabilization Buffer consequently. Next, cells were stained with FITC‐MAb1 and chromogenic anti‐FITC secondary reagent, suspended in fluorescence activated cell sorting (FACS) buffer to perform the flow cytometry assay.

### 2.9 Statistical analysis

Analysis of data were performed using IBM SPSS Statistics. The Student's *t*‐test was used to compare groups, unless stated otherwise, GraphPad Prism was employed to analyze data, presented as mean ± standard error of the mean. A *p* < 0.05 was considered as statistical significance.

## 3 RESULTS

### 3.1 Hypoxia‐related pathways are associated with responses to Sorafenib in HCC patients

**FIGURE 1:**
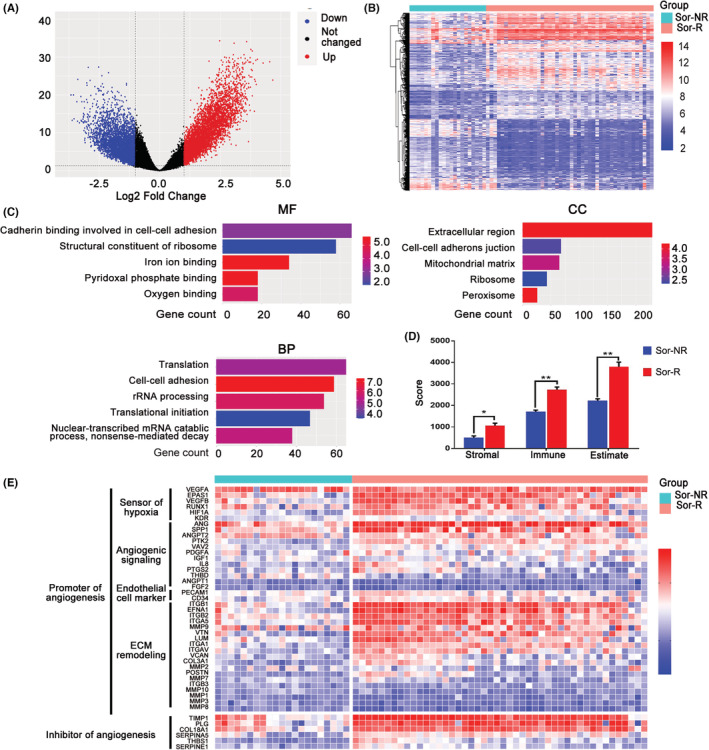
DEGs and DE‐pathways between Sor‐R and Sor‐NR HCC patients. (A) Volcano plot of DEGs between group of Sor‐R and Sor‐NR HCC patients. Black dots, blue dots and red dots represent unchanged, down‐regulated, and up‐regulated genes in Sor‐R group compared to Sor‐NR group, respectively. (B) Heatmap of DEGs between group of Sor‐R and Sor‐NR HCC patients. (C) GO functional enrichment analysis of DEGs between group of Sor‐R and Sor‐NR HCC patients. (D) The Stromal score, Immune score, and Estimate score between group of Sor‐NR and Sor‐R HCC patients. (E) Heatmap of genes involved in angiogenesis between group of Sor‐R and Sor‐NR HCC patients. DEGs, differentially expressed genes; HCC, hepatocellular carcinoma; Sor‐NR, Sorafenib‐non‐resistant; Sor‐R, Sorafenib‐resistant. *: *p*〈0.05; **: *p*〈0.01.

Sixty‐seven HCC patients (GSE109211) were grouped into Sor‐R (46 patients) and Sor‐NR (21 patients) based on their responses to Sorafenib treatment, transcriptomic data of whom were downloaded for analysis.^11^ Using the thresholds of *p* < 0.05 and absolute log2FoldChange >1, a total of 29,377 differential expression genes (DEGs) between Sor‐R and Sor‐NR groups were identified and presented in volcano plots (Figure 1A). These DEGs also displayed distinct distribution in each group as exhibited in heatmap (Figure 1B). To identify the biological functions of those DEGs, we performed gene ontology (GO) analysis with those DEGs. The biological functional and molecular pathway enrichment analysis showed that difference in cell–cell adhesion may contributed to various responses to Sorafenib treatment (Figure 1C). Correspondingly, result of ESTIMATE algorithm showed higher score of tumor purity in Sor‐R compared to Sor‐NR group, indicating a more complicated tumoral microenvironment in resistant process (Figure 1D). Because vascular system is necessary for tumor growth and immune cell filtration, a gene panel involved in promoting or inhibiting vessel formation was selected and presented (Figure 1E). As expected, genes involved in vessel formation (e.g., Sensor of hypoxia, ECM remodeling) were greatly stronger in Sor‐R than Sor‐NR group, suggesting more robust angiogenic cascade. However, markers of endothelial originated vessel (PECAM1 and CD34) were constant, followed with elevated inhibitor of angiogenesis (TIMP1, PLG).

### 3.2 ITGA5 and ITGB1 were positively correlated to hypoxia in HCC patients

**FIGURE 2:**
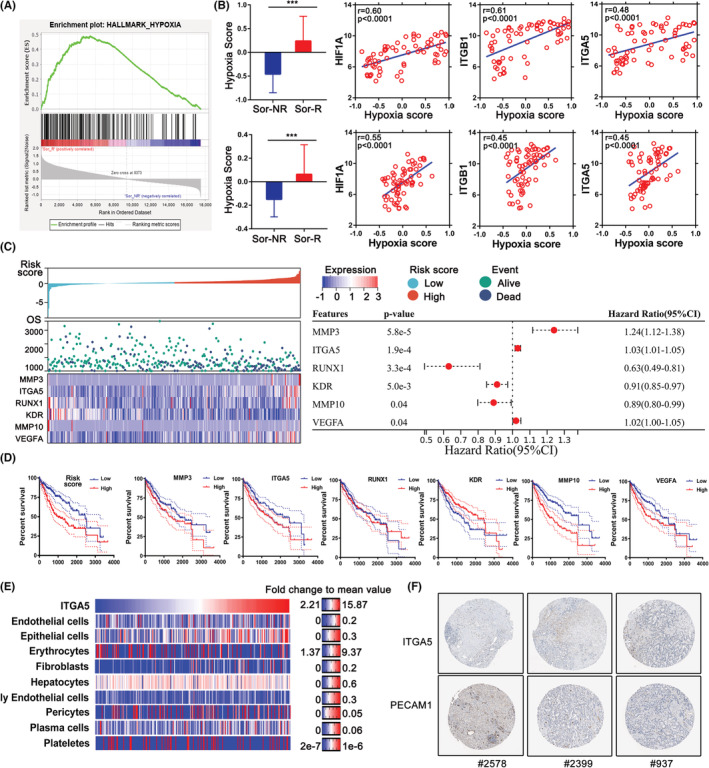
Evaluation of hypoxic character in Sor‐R and Sor‐NR HCC patients from TCGA database. (A) GSEA result between Sor‐R and Sor‐NR HCC patients by using Hallmark Hypoxia gene set. (B) Buffa's (upper) and Winter's (lower) hypoxia model was used to calculate hypoxia score of Sor‐R and Sor‐NR HCC patients. (B) Correlation between hypoxia score with ITGA5 or ITGB1 in Sor‐R or Sor‐NR HCC patients. (C) OS analyses by single marker cut‐offs optimized using multivariate Cox regression. (D) Kaplan–Meier method was used to compute overall survival of HCC patients from TCGA database with distinct expression of candidate genes. (E) Heatmap of ITGA5 level and vessel‐related cells in using xCell algorithm in TCGA‐LIHC cohort. (F) Protein level of ITGA5 and PECAM1 in HCC tissue which were downloaded from the Human Protein Atlas. GSEA, gene set enrichment analysis; HCC, hepatocellular carcinoma; ITGA5, integrins A5; ITGB1, integrins B1; Sor‐R, Sorafenib‐resistant; Sor‐NR, Sorafenib‐non‐resistant; TCGA, the cancer genome atlas. ***: *p*〈0.001.

Given the impact of hypoxia on cell adhesion and tumor malignancy, we focused on the difference of hypoxia level between Sor‐R and Sor‐NR patients. Firstly, we performed GSEA by using gene sets of hypoxia, and observed altered enrichment of hypoxic genes between those two groups (Figure 2A). Next, we calculated hypoxia scores by using Buffa's or Winter's hypoxia signatures as described in methods. In coincident with our expectation, the hypoxia level was prominently higher in Sor‐R group (Figure 2B). Moreover, level of hypoxia is correlated with major vessel genes, including HIF1A, ITGA5, and ITGB1 (Figure 2B). Next, we sought to determine a gene cluster responsible for Sorafenib‐resistance by performing the Cox proportional hazards model with elastic net regression and growing random forests. A six‐gene signature was filtered as a classifier. Risk score and coeffs value of multivariable Cox regression of each patient were computed with expression of those genes (Figure 2C). The formula to calculate Risk score is as following: Risk score = 0.2 × MMP3 + 0.023 × ITGA5−0.127 × RUNX1−0.075 × KDR‐0103 × MMP10 + 0.025 × VEGFA. According to this formula, survival rates of risk score or each candidate gene were presented, almost the genes of interest emerged as significant predictors of worse overall survival (OS) (Figure 2D). Based on above results, we concluded ITGA5 may participate in hypoxia‐related Sorafenib resistance in HCC patients. Along with elevated expression of ITGA5, we observed an increase of epithelial cells but slightly reduced erythrocytes and pericytes (Figure 2E). To be noted, there is a trade‐off between ITGA5 and PECAM1 in HCC tissue, but the samples can be accessed are too limited to make it more confirmed (Figure 2F). Those results suggested that angiogenetic‐related genes possess prognostic power for response to Sorafenib treatment in HCC patients, and we hypothesized that hypoxic character may be affected by ITGA5 and corresponding β subunit, especially ITGB1 (Figure S1).

### 3.3 ITGA5 and ITGB1 promoted Sorafenib‐resistance in HCC

**FIGURE 3:**
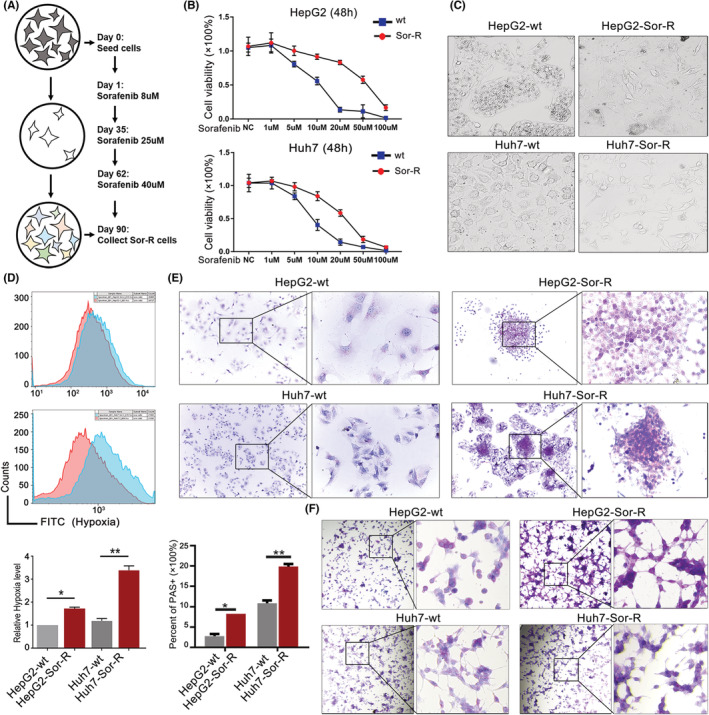
ITGA5 and ITGB1 boosted VM formation. (A) Workflow diagram of constructing Sor‐R cell lines. (B) Confirmation of tolerance to Sorafenib in Sor‐R cells by cell killing assay. (C) Observation of morphological change in HCC cells resistant to Sorafenib compared to original cells. (D) Hypoxia level detection in Sorafenib‐resistant HepG2/Huh7 cells via flow cytometry assay. The red curve represents control, and the blue curves represent the Sorafenib‐resistant HepG2/Huh7 cells. (E) Detection of VM structure in wildtype and Sor‐R cells on glass slides by PAS staining. (F) Detection of VM structure in wildtype and Sor‐R cells on 3D scaffolds by PAS staining. HCC, hepatocellular carcinoma; ITGA5, integrins A5; ITGB1, integrins B1; PAS, periodic acid schiff; Sor‐R, Sorafenib‐resistant; VM, vasculogenic mimicry. *: *p*〈0.05 ; **: *p*〈0.01.

To confirm the role of ITGA5 and ITGB1 in Sorafenib resistance, we generated the Sor‐R cell lines using HepG2 and Huh7 cells (named with HepG2‐Sor‐R and Huh7‐Sor‐R) treated with accelerating concentration of Sorafenib (Figure 3A). Successful establishment of Sor‐R cell lines were confirmed by CCK8 assay (Figure 3B). Obvious morphological change including strengthened pseudopodia formation were observed in Sor‐R cells compared to wildtype cells (Figure 3C). The hypoxyprobe was utilized to determine the change of oxygen concentration, which can form detectable adducts with thiol groups in biological components in cellular hypoxic area. There was increased hypoxia signal detected in Sor‐R cells, indicating a robust anoxic environment was accompanied with Sorafenib resistance (Figure 3D). To study the changes of VM formation after acquiring resistance to Sorafenib, we performed PAS staining in cells grown on glass slides or 3D collagen scaffold separately. Both results showed an augmentation of VM structure in Sor‐R cells compared to wildtype cells, suggesting that VM contribute to Sorafenib resistance in HCC cells (Figure 3E,F).

**FIGURE 4:**
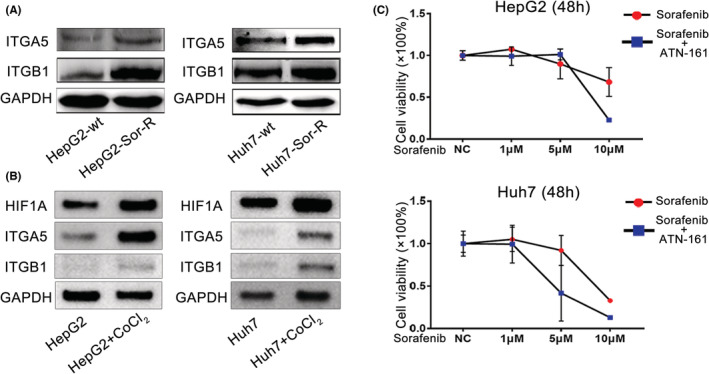
ITGA5 and ITGB1 reduced sensitivity to Sorafenib. (A) Measurement of protein levels of ITGA5 and ITGB1 in wildtype and HepG2/Huh7 Sor‐R cells. (B) Changes of ITGA5 and ITGB1 in HepG2/Huh7 cells treated with 150 μM CoCl_2_. (C) Measurement of sensitivity to Sorafenib and Sorafenib plus integrin inhibitor (ATN‐161) in HepG2 and Huh7 cells. ITGA5, integrins A5; ITGB1, integrins B1; Sor‐R, Sorafenib‐resistant.

Next, we investigated the role of ITGA5 and ITGB1 in establishing Sorafenib resistance. Upregulated expression of ITGA5 and ITGB1 were observed in Sor‐R cells than wildtype cells (Figure 4A). To explore whether hypoxia participates in inducing ITGA5 and ITGB1 expression, a hypoxia stimulatory agent, CoCl_2_ was used. After being treated with 150 μM CoCl_2_ for 3 days, expression of ITGA5 and ITGB1 were both upregulated, together with increased HIF1A (Figure 4B). To annotate the role of ITGA5 and ITGB1in mediating Sorafenib resistance, a specific integrin antagonist, ATN‐161, was chosen to block them. As expected, ATN‐161 enhanced cytotoxicity of Sorafenib on HepG2 and Huh7 cells, especially in condition of high‐dosage treatment (Figure 4C). These observations indicated that ITGA5 and ITGB1 were upregulated during process of Sorafenib resistance, accompanied by hypoxic microenvironment and VM formation.

**FIGURE 5:**
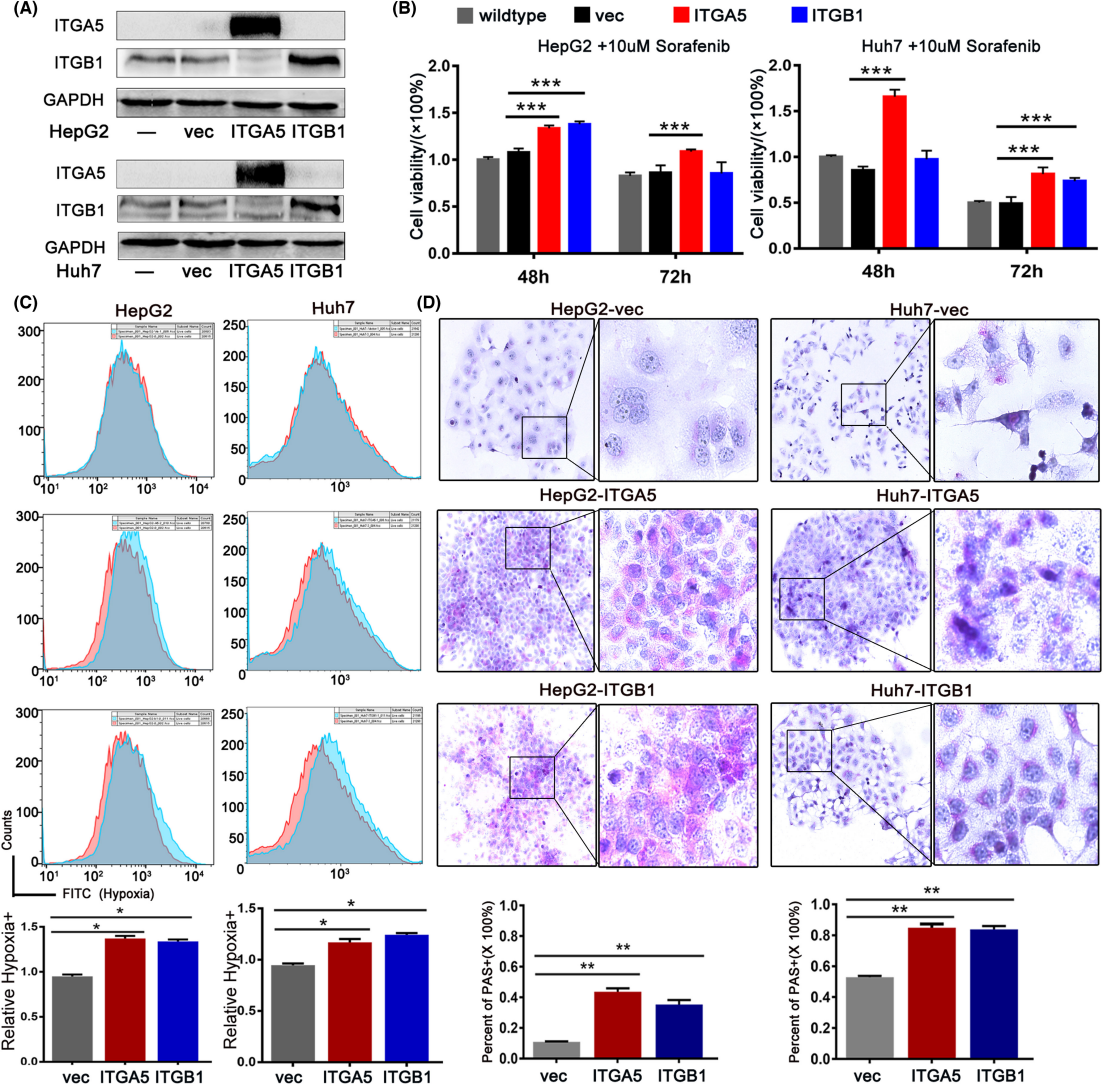
ITGA5 and ITGB1 promoted Sorafenib resistance by enhancing hypoxia and VM formation. (A) Confirmation of ITGA5 or ITGB1 overexpression cells by Western blot. (B) Evaluation of tolerance to Sorafenib in control and ITGA5 or ITGB1 overexpressing cells by cell killing assay. (C) Detection of hypoxia level in control and ITGA5 or ITGB1 overexpressing cells by flow cytometry assay. (D) Quantification of VM structure in control and ITGA5 or ITGB1 overexpressing cells by PAS staining. Microphotographs were collected and uploaded into ImageJ software for quantification. ITGA5, integrins A5; ITGB1, integrins B1; PAS, periodic acid schiff; VM, vasculogenic mimicry. *: *p*〈0.05 ; **: *p*〈0.01; ***: *p*〈0.001.

To verify role of ITGA5 and ITGB1 in cellular hypoxia and VM formation, we constructed ITGA5 or ITGB1 overexpressing cells by using a lentiviral system as verified by Western blot (Figure 5A). With ITGA5 or ITGB1 overexpressed, more cells survived from Sorafenib treatment (10 μM), manifesting that overexpressed ITGA5 or ITGB1 enhanced tolerance to Sorafenib (Figure 5B). In accompany with that, increased hypoxia level and VM structure formation were observed in cells overexpressed with ITGA5 or ITGB1 (Figure 5C,D). Collectively, ITGA5 and ITGB1 induced hypoxia and VM formation, which may play an important role in Sorafenib resistance.

## 4 DISCUSSION

Resistance to Sorafenib in HCC patients restricted their survival benefit severely. Several major steps in resistance process have been elaborated, including drug metabolism, oncogenic driver gene, loss of drug target, and so on. At first, mutation or alteration on particular enzymes may contribute to reduced bioavailability of Sorafenib, as that metabolism of Sorafenib occurs primarily in the liver via cytochrome P450 (CYP) 3A4 and uridine diphosphate glucuronosyltransferase (UGT)1A9.^14^ Drug‐host or drug–drug interaction has been proved to reduce drug intake or enhanced drug export, thereby limiting utilization rate of Sorafenib.^15^ Secondly, the high heterogeneity of HCC tumor and microenvironment also set an obstacle for drug effect. During Sorafenib treatment, immune‐suppressive microenvironment may be aggravated, protecting tumor from immune eradication. Indeed, augmentation of cytotoxic T cells was associated with superior survival, indicating that sufficient immune activation is required for response to Sorafenib treatment in HCC treatment.^16^

Apart from the above reasons, heterogeneity of vascular system is responsible for blocking molecular target of Sorafenib, making the issue of resistance more complicated. As a common property of solid tumors, cells in central region are facing with an exceeding hypoxic microenvironment and starvation. To meet the demands of oxygen and nutrients, hypoxia tensions induce robust neovascularization by stress ECs to differentiation. Even more, therapeutic options like transarterial chemoembolization, or multiple chemotherapy drugs especially angiogenesis inhibitors are reported to interfere existing blood vessels, leading to severer ischemia, hypoxia inside tumor tissue.^17^ HIF‐1 together with HIF‐1 regulated genes are considered to play a pivotal role in perceiving oxygen change. Under hypoxic cellular conditions, diminished degraded HIF‐1α conjugates with the stable HIF‐1β to dimer into HIF‐1.^18^ Heterodimerized HIF‐1 can bind with hypoxia response elements in the promoter region of target gene, overactivating tumor‐associated signaling pathway like MAPK, PI3K, enabling tumor cells to be more invasive, and more proliferative.^19^

Since being firstly reported by Maniotis et al. in 1999, hypoxia‐responsive ability of VM in solid cancer has been unearthed by accumulating research.^20^ Under the hypoxic condition, VM or mosaic vessels can be stimulated from tumor cells to replenish incapable activity of typical ECs originated vessels. For example, treating renal carcinoma cells with Sunitinib, an anti‐angiogenetic drug, blocked VM function efficiently in the initial time. However, VM recovered quickly from Sunitinib, followed with tumor resistance and a more aggressive phenotype.^21^ As described above, tumor cell‐origin endowed VM with more aggressive and metastatic characteristics compared to routine vessels. Those VM positive area are always accompanied with pseudopodia and invadopodia production. Aggressive pseudopodia can recruit matrix metalloproteinases (MMPs) to the leading edge to degrade basement membrane ECM, promoting tumor invasion and metastasis.^22^ Additionally, VM phenomenon enables tumor cells exposed to the blood stream directly, making them easily transferred with blood. VMs are tumor‐specific but absent in normal liver tissue, associated with more rapid posttransplant recurrence.^23^ Quantity of VM was positively associated with a high tumor grade, invasion and metastasis, and short survival in HCC patients.

Even though the mechanism of VM formation remains suspending, ECM remodeling and EMT are certainly involved in this process, which are under regulation of activated NOTCH or PI3K pathways.^24^ Original ECM structure containing collagens and fibronectins can be degraded and reshaped by a batch of MMPs, providing space for tubular structure construction. For example, both expression and activity of MMP‐2 and MMP‐9 were found enriched in the VM‐positive lesions gastrointestinal stromal tumors. Once activated, MMP2 drills and lysis ECM components like Ln5γ2 (laminin) and in the early phase of VM ducts formation.^25^ Besides with MMPs, other EMT‐related enzymes or factors also attribute to ECM components organization, modified cell polarity and VM development. During this process, integrin can facilitate metastasis and influence the malignant phenotype in several cancers.^26^ Members of integrin are reported to affect invasion by inducing EMT in HCC.^27^ Pro‐angiogenic role of integrin especially ITGA5/ITGB1 dimer (naming α5β1) has been observed, as overexpressed α5β1 are enriched in CD31^+^ vessel structure. To be noted, these α5β1 mediated vessels are different from normal VEGF‐dependent blood vessels.^28^ When overexpressed in cancer cells, their pro‐angiogenic ability endow cancer cells to form VM and display vessel‐like function. ITGB1‐knockout cancer cells failed to form VM network, while reintroduction of ITGB1 rescued VM formation in those cells.^29^ Physical interaction of ITGA5 and ITGB1 with ligands is essential in cell adhesion, signaling pathways and cytoskeletal organization and force generation. This bridge of integrin and their adhesion protein ligands, such as collagens, thrombospondin and laminin, is mainly through recognizing specific arginine‐glycine‐aspartate (Arg‐Gly‐Asp, RGD) motif.^30^

Considering that integrin‐mediated VM is essential in tumor invasion and drug resistance, drugs targeting on integrin are proposed and are under laboratorial or preclinical investigation. ATN‐161 adopted in this study, is a fibronectin derived non‐competitive α5β1 inhibitor with antiangiogenic and antimetastatic ability. In a phase I clinical trial, patients showed tolerance to ATN‐161as well as improved therapeutic outcome.^31^ Cilengitide is a cyclic RGD peptide with potential antineoplastic activity, which can specifically bind to and inhibit the activities of several integrins (αVβ3, αVβ5, α5β1). Currently, Cilengitide is adopted in a phase III clinical trial for treatment of glioblastomas and in a phase II trial for other types of tumors.^32^

In summary, our study provided an explanation for that antiangiogenic drug do not meet the therapeutic effect as expected: mostly because of the appearance of hypoxia‐induced VM. Formation of VM in HCC is under the control of ITGA5 and ITGB1, while targeting them might be a promising therapeutic choice for HCC patients resistant to Sorafenib.

## AUTHOR CONTRIBUTIONS

Ying Shi, Jin Shang: Design of the work; Ying Shi, Yan Li, Zilong Zhang: Experiment performance, data collection; Jin Shang, Deyuan Zhong: Data analysis and interpretation, drafting the article; Qinyan Yang, Chunyou Lai, Tianhang Feng: Critical revision of the article; Xiaolun Huang, Ying Shi: Final approval of the version to be published. All authors reviewed the manuscript.

## FUNDING INFORMATION

This work was supported by the Sichuan Science and Technology Program under Grant 2021YFH0187; Fundamental Research Funds for the Central Universities under Grant ZYGX2020KYQD002 and ZYGX2021YGCX018.

## CONFLICT OF INTEREST

All authors report that there are no competing interests to declare.

## Supporting information


Figure S1

## Data Availability

The public data used in this manuscript were downloaded in GEO, TCGA, THPA databases as mentioned in Materials and methods.

## References

[1] Sung HA‐O , Ferlay J , Siegel RA‐O , et al. Global cancer statistics 2020: GLOBOCAN estimates of incidence and mortality worldwide for 36 cancers in 185 countries. CA Cancer J Clin. 2021;71:209‐249. 33538338 10.3322/caac.21660

[2] Lu J , Zhang XP , Zhong BY , et al. Management of patients with hepatocellular carcinoma and portal vein tumour thrombosis: comparing east and west. Lancet Gastroenterol Hepatol. 2019;4(9):721‐730. 31387735 10.1016/S2468-1253(19)30178-5

[3] Llovet JM , Ricci S , Mazzaferro V , et al. Sorafenib in advanced hepatocellular carcinoma. N Engl J Med. 2008;359(4):378‐390. 18650514 10.1056/NEJMoa0708857

[4] Ford R , Schwartz L , Dancey J , et al. Lessons learned from independent central review. Eur J Cancer. 2009;45(2):268‐274. 19101138 10.1016/j.ejca.2008.10.031

[5] Qian CN , Pezzella F . Tumor vasculature: a sally port for inhibiting cancer cell spreading. Cancer Commun (Lond). 2018;38(1):52. 30075743 10.1186/s40880-018-0322-z PMC6076415

[6] Mao Y , Zhu L , Huang Z , et al. Stem‐like tumor cells involved in heterogeneous vasculogenesis in breast cancer. Endocr Relat Cancer. 2020;27(1):23‐39. 31705798 10.1530/ERC-19-0054

[7] Luo Q , Wang J , Zhao W , et al. Vasculogenic mimicry in carcinogenesis and clinical applications. J Hematol Oncol. 2020;13(1):19. 32169087 10.1186/s13045-020-00858-6 PMC7071697

[8] van der Schaft DW , Seftor RE , Seftor EA , et al. Effects of angiogenesis inhibitors on vascular network formation by human endothelial and melanoma cells. J Natl Cancer Inst. 2004;96(19):1473‐1477. 15467037 10.1093/jnci/djh267

[9] Semenza GL . Hypoxia‐inducible factors: mediators of cancer progression and targets for cancer therapy. Trends Pharmacol Sci. 2012;33(4):207‐214. 22398146 10.1016/j.tips.2012.01.005 PMC3437546

[10] Costa P , Parsons M . New insights into the dynamics of cell adhesions. Int Rev Cell Mol Biol. 2010;283:57‐91. 20801418 10.1016/S1937-6448(10)83002-3

[11] Pinyol R , Montal R , Bassaganyas L , et al. Molecular predictors of prevention of recurrence in HCC with sorafenib as adjuvant treatment and prognostic factors in the phase 3 STORM trial. Gut. 2019;68(6):1065‐1075. 30108162 10.1136/gutjnl-2018-316408 PMC6580745

[12] Buffa FM , Harris AL , West CM , Miller CJ . Large meta‐analysis of multiple cancers reveals a common, compact and highly prognostic hypoxia metagene. Br J Cancer. 2010;102(2):428‐435. 20087356 10.1038/sj.bjc.6605450 PMC2816644

[13] Wu HB , Yang S , Weng HY , et al. Autophagy‐induced KDR/VEGFR‐2 activation promotes the formation of vasculogenic mimicry by glioma stem cells. Autophagy. 2017;13(9):1528‐1542. 28812437 10.1080/15548627.2017.1336277 PMC5612353

[14] Marin JJ , Romero MR , Briz O . Molecular bases of liver cancer refractoriness to pharmacological treatment. Curr Med Chem. 2010;17(8):709‐740. 20088759 10.2174/092986710790514462

[15] Keating GM , Santoro A . Sorafenib: a review of its use in advanced hepatocellular carcinoma. Drugs. 2009;69(2):223‐240. 19228077 10.2165/00003495-200969020-00006

[16] Kalathil SG , Hutson A , Barbi J , Iyer R , Thanavala Y . Augmentation of IFN‐gamma^+^ CD8^+^ T cell responses correlates with survival of HCC patients on sorafenib therapy. JCI Insight. 2019;4(15):e130116. 31391334 10.1172/jci.insight.130116 PMC6693832

[17] Liu K , Min XL , Peng J , Yang K , Yang L , Zhang XM . The changes of HIF‐1alpha and VEGF expression after TACE in patients with hepatocellular carcinoma. J Clin Med Res. 2016;8(4):297‐302. 26985249 10.14740/jocmr2496w PMC4780492

[18] Jiang BH , Rue E , Wang GL , Roe R , Semenza GL . Dimerization, DNA binding, and transactivation properties of hypoxia‐inducible factor 1. J Biol Chem. 1996;271(30):17771‐17778. 8663540 10.1074/jbc.271.30.17771

[19] Liu Z , Wang Y , Dou C , et al. Hypoxia‐induced up‐regulation of VASP promotes invasiveness and metastasis of hepatocellular carcinoma. Theranostics. 2018;8(17):4649‐4663. 30279729 10.7150/thno.26789 PMC6160773

[20] Maniotis AJ , Folberg R , Hess A , et al. Vascular channel formation by human melanoma cells in vivo and in vitro: vasculogenic mimicry. Am J Pathol. 1999;155(3):739‐752. 10487832 10.1016/S0002-9440(10)65173-5 PMC1866899

[21] Serova M , Tijeras‐Raballand A , Dos Santos C , et al. Everolimus affects vasculogenic mimicry in renal carcinoma resistant to sunitinib. Oncotarget. 2016;7(25):38467‐38486. 27509260 10.18632/oncotarget.9542 PMC5122404

[22] Stylli SS , Kaye AH , Lock P . Invadopodia: at the cutting edge of tumour invasion. J Clin Neurosci. 2008;15(7):725‐737. 18468901 10.1016/j.jocn.2008.03.003

[23] Guzman G , Cotler SJ , Lin AY , Maniotis AJ , Folberg R . A pilot study of vasculogenic mimicry immunohistochemical expression in hepatocellular carcinoma. Arch Pathol Lab Med. 2007;131(12):1776‐1781. 18081435 10.5858/2007-131-1776-apsovm PMC2617786

[24] Wei X , Chen Y , Jiang X , et al. Mechanisms of vasculogenic mimicry in hypoxic tumor microenvironments. Mol Cancer. 2021;20(1):7. 33397409 10.1186/s12943-020-01288-1 PMC7784348

[25] Hess AR , Seftor EA , Seftor RE , Hendrix MJ . Phosphoinositide 3‐kinase regulates membrane type 1‐matrix metalloproteinase (MMP) and MMP‐2 activity during melanoma cell vasculogenic mimicry. Cancer Res. 2003;63(16):4757‐4762. 12941789

[26] Guo W , Giancotti FG . Integrin signalling during tumour progression. Nat Rev Mol Cell Biol. 2004;5(10):816‐826. 15459662 10.1038/nrm1490

[27] Ke AW , Shi GM , Zhou J , et al. CD151 amplifies signaling by integrin alpha6beta1 to PI3K and induces the epithelial‐mesenchymal transition in HCC cells. Gastroenterology. 2011;140(5):1629‐1641.e1615. 21320503 10.1053/j.gastro.2011.02.008

[28] Kim S , Bell K , Mousa SA , Varner JA . Regulation of angiogenesis in vivo by ligation of integrin alpha5beta1 with the central cell‐binding domain of fibronectin. Am J Pathol. 2000;156(4):1345‐1362. 10751360 10.1016/s0002-9440(10)65005-5 PMC1876892

[29] Kawahara R , Niwa Y , Simizu S . Integrin beta1 is an essential factor in vasculogenic mimicry of human cancer cells. Cancer Sci. 2018;109(8):2490‐2496. 29900640 10.1111/cas.13693 PMC6113431

[30] Ruoslahti E , Pierschbacher MD . New perspectives in cell adhesion: RGD and integrins. Science. 1987;238(4826):491‐497. 2821619 10.1126/science.2821619

[31] Cianfrocca ME , Kimmel KA , Gallo J , et al. Phase 1 trial of the antiangiogenic peptide ATN‐161 (Ac‐PHSCN‐NH(2)), a beta integrin antagonist, in patients with solid tumours. Br J Cancer. 2006;94(11):1621‐1626. 16705310 10.1038/sj.bjc.6603171 PMC2361324

[32] Edwards DN , Salmeron K , Lukins DE , Trout AL , Fraser JF , Bix GJ . Integrin α5β1 inhibition by ATN‐161 reduces neuroinflammation and is neuroprotective in ischemic stroke. J Cereb Blood Flow Metab. 2020;40(8):1695‐1708. 31575337 10.1177/0271678X19880161 PMC7370357

