# Supplementary figures and images for: ITGA5 and ITGB1 contribute to Sorafenib resistance by promoting vasculogenic mimicry formation in hepatocellular carcinoma
Source: Cancer Med. 2022 Aug 10;12(3):3786–96. doi: 10.1002/cam4.5110 (PMC9939139; doi:10.1002/cam4.5110)

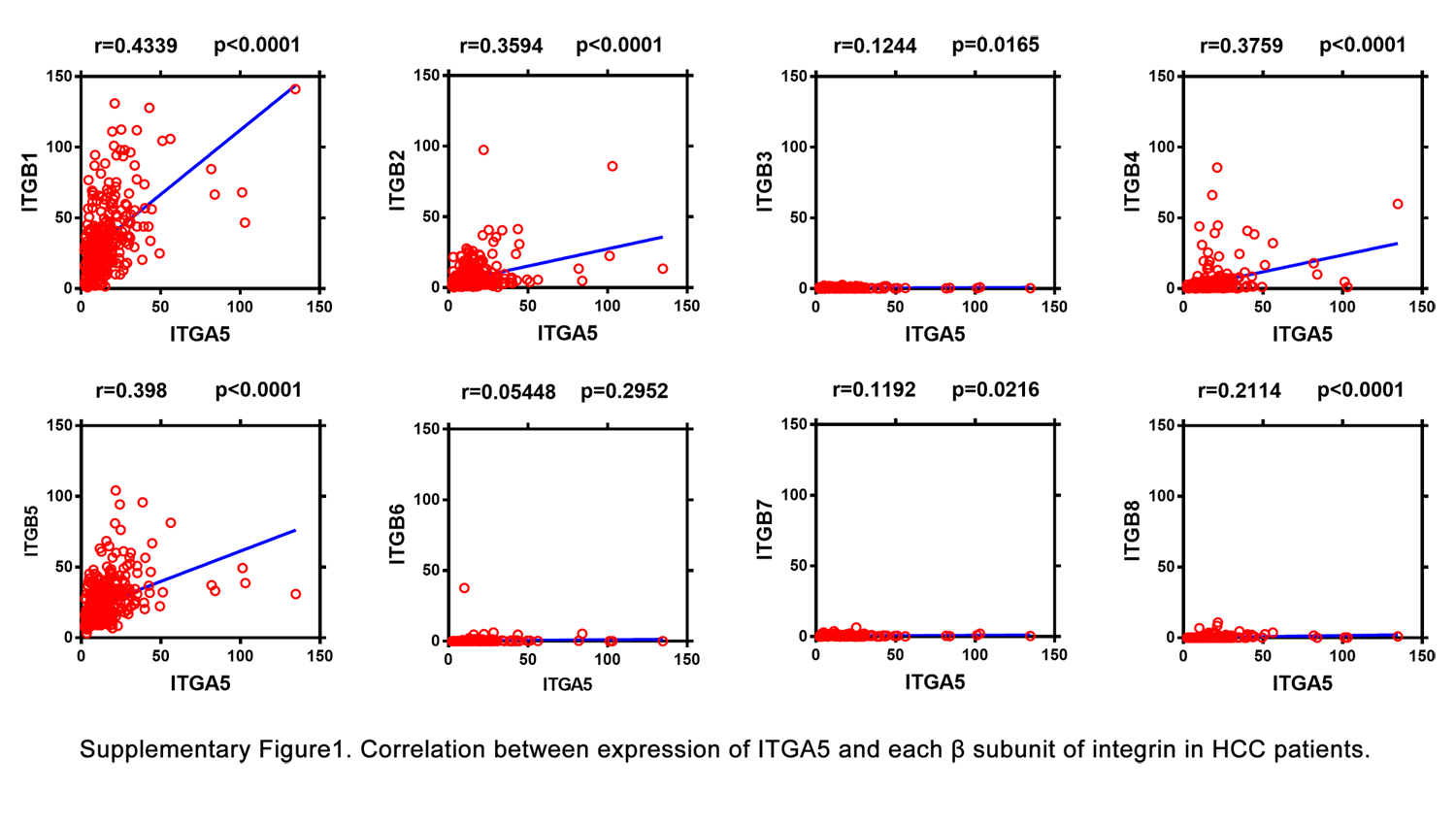

Supplement: Supplementary file 1 — Figure S1 [file CAM4-12-3786-s001.tif]
